# Exercise, cognition, and affective disorders

**DOI:** 10.3389/fspor.2026.1801197

**Published:** 2026-05-07

**Authors:** Alejandro Tapia-de Jesús, Mario Humberto Buenrostro-Jáuregui, César I. Ayala-Guzmán, Jesús Mata-Luévanos

**Affiliations:** 1 Department of Health, Universidad Iberoamericana, Mexico, Mexico; 2 Laboratory of Neuroscience, Department of Psychology, Universidad Iberoamericana, Mexico, Mexico; 3 Laboratory of Integration in Motivation, Emotion and Neuroscience, Facultad de Medicina y Ciencias Biomédicas, Universidad Autónoma de Chihuahua, Chihuahua, Mexico

**Keywords:** cognitive function, depression and anxiety, inflammation, neurobiological resilience, neuroplasticity, physical exercise, stress regulation (HPA axis)

## Abstract

Anxiety and depressive disorders are major contributors to global disability and are frequently accompanied by cognitive impairment, stress-system dysregulation, and cardiometabolic comorbidity. Although pharmacological and psychotherapeutic treatments remain central, limited access, partial response, and persistent residual symptoms highlight the need for integrative and scalable interventions. This narrative review synthesizes evidence on physical activity and structured exercise as systemic biological interventions for depression and anxiety, integrating clinical findings with mechanistic pathways linking peripheral physiological adaptations to brain, cognitive, and emotional outcomes. We reviewed meta-analyses, randomized controlled trials, and mechanistic studies examining how exercise-induced mechanical, metabolic, endocrine, immune, and neurochemical signals converge on neural plasticity, stress regulation, and executive functioning. Across populations, habitual physical activity is consistently associated with lower depression risk, following nonlinear dose–response patterns, while exercise interventions show robust effects for depressive symptoms and smaller but consistent benefits for anxiety. Mechanistically, exercise acts as a predictable and dosable physiological stressor that improves stress-axis regulation, reduces low-grade systemic inflammation, modulates neurochemical systems, and promotes neuroplasticity in frontolimbic circuits. These adaptations are proposed to support improvements in executive functions and affective cognition, reducing rumination and strengthening neurobiological resilience. We propose cognition as a functional node linking systemic exercise-induced adaptations to clinical outcomes, positioning exercise not only as a symptom-reduction strategy but as a transdiagnostic intervention targeting core pathophysiological processes in depression and anxiety. This integrative framework supports the incorporation of exercise into multimodal mental health care and highlights priorities for individualized prescription and future mechanism-informed research.

## 1 Introduction

Over the past decades, anxiety and depressive disorders have become leading causes of disability worldwide, with substantial impacts on individual health, productivity, and health-care systems. This burden increased markedly during the COVID-19 pandemic, underscoring these conditions as a public health priority and highlighting the magnitude of their global impact (1). Beyond their high prevalence, anxiety and depression are associated with increased risk of cardiovascular, metabolic, and neurodegenerative diseases, as well as higher premature mortality (2, 3).

Although pharmacological and psychotherapeutic treatments remain foundational in clinical care, a significant gap persists between need and effective access, alongside a substantial proportion of patients who show partial response, frequent relapse, or intolerance to conventional therapies. These limitations have motivated a critical reassessment of traditional explanatory models, favoring broader frameworks that integrate dysregulation of stress systems, low-grade systemic inflammation, impaired neuroplasticity, and cognitive dysfunction as central components of the pathophysiology of anxiety and depression (3).

In parallel, contemporary social environments are characterized by sustained increases in chronic stress, physical inactivity, and continuous exposure to the cognitive and emotional demands of digital life. These factors interact with pre-existing biological vulnerabilities and contribute to both the emergence and the persistence of affective symptoms. In this context, interest has grown in therapeutic interventions that not only reduce symptom burden but also strengthen adaptive capacity to stress and promote long-term resilience.

Within this framework, physical activity and structured exercise have emerged as interventions with particularly relevant therapeutic potential in mental health. Initially viewed as complementary behavioral strategies, they are now recognized as systemic biological modulators capable of inducing robust adaptations through neuroendocrine, immunoinflammatory, and neurotrophic pathways, which are directly implicated in mood regulation, cognitive function, and stress tolerance (4, 5). Recent clinical evidence supports their efficacy both as stand-alone interventions and as adjunctive approaches—and, in specific contexts, as therapeutic alternatives for mild-to-moderate depression and anxiety.

From a mechanistic perspective, exercise can be understood as the introduction of a controlled physiological stressor that engages musculoskeletal, metabolic, and endocrine signaling pathways, which are thought to converge on functional and structural brain changes. These adaptations include modulation of the hypothalamic–pituitary–adrenal axis, reductions in low-grade systemic inflammation, regulation of neurochemical systems, and reinforcement of neuroplasticity in brain regions critical for emotional regulation and executive functioning (3).

**Figure 1:**
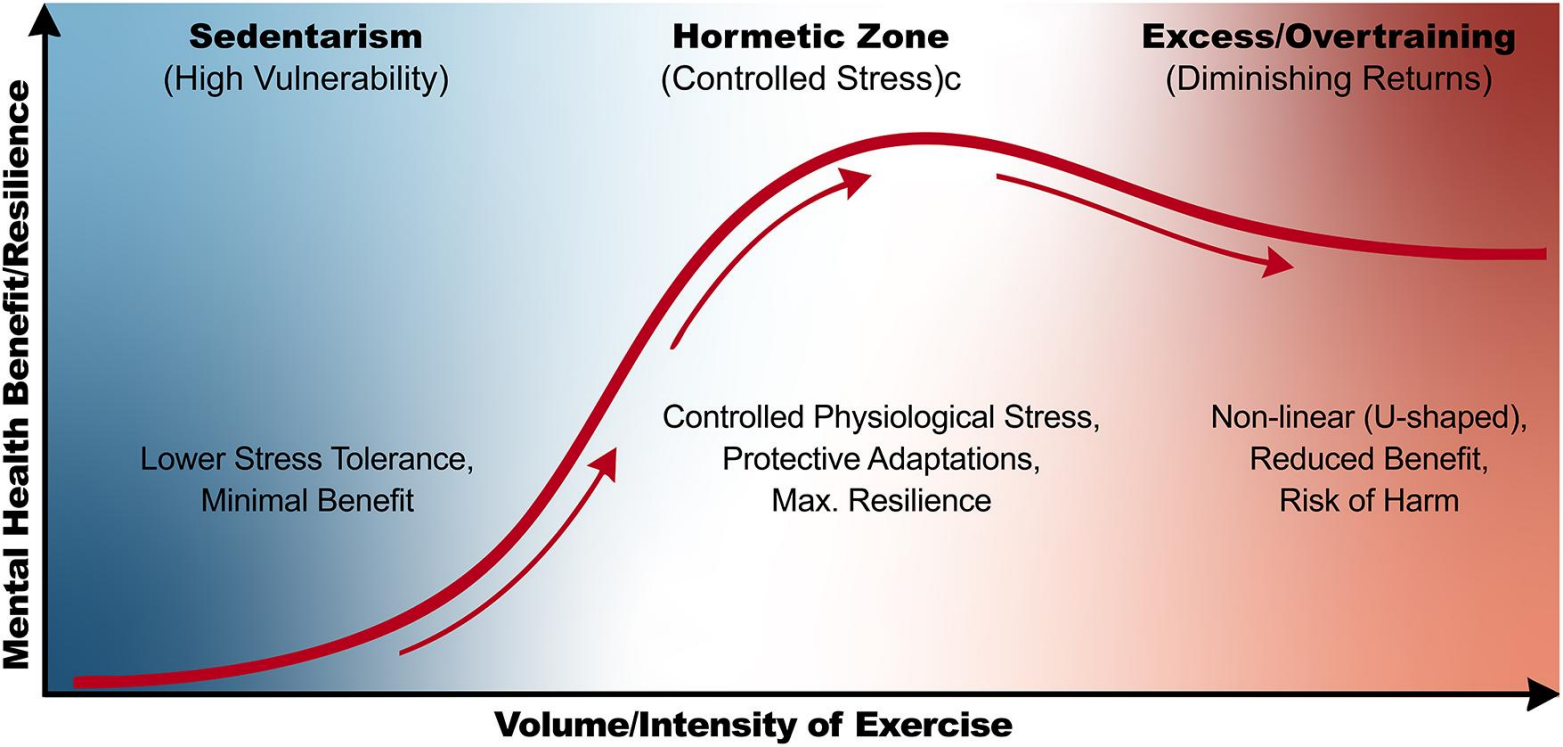
The hormesis ncurve: dose-response in mental health. Schematic illustration of the hormetic dose–response relationship between exercise and mental health, showing that moderate levels of physical activity confer maximal psychological benefits, whereas both sedentary behavior and excessive exercise are associated with diminished or suboptimal mental health outcomes.

A key conceptual element for understanding the therapeutic potential of exercise is acknowledging that its effects depend on dose and progressive titration of the stimulus. When appropriately prescribed, exercise operates within a hormetic framework in which repeated exposure to moderate biological challenges promotes protective adaptations and strengthens neuronal and metabolic resilience (6, 7). Figure 1 illustrates the hormetic dose–response relationship, in which moderate exercise elicits maximal mental health benefits, whereas both sedentarism and excessive exercise result in suboptimal outcomes. This integrative view positions exercise not only as a symptom-reduction strategy but also as an intervention capable of modulating transdiagnostic pathophysiological processes involved in anxiety and depression.

Unlike reviews focused exclusively on affective symptoms, the present review integrates mechanical and peripheral adaptations to exercise with cognitive and resilience-related changes, proposing cognition as a functional node linking physiological mechanisms to clinical outcomes.

Accordingly, this review advances an integrative framework to explain how physical activity and exercise, through peripheral and systemic adaptations, translate into brain, cognitive, and emotional changes relevant to anxiety and depression, and how these mechanisms support the growing incorporation of exercise into contemporary clinical practice.

## 2 Methods

We conducted an integrative narrative review to analyze and synthesize the scientific evidence on the effects of physical activity and exercise on anxiety and depression, with particular emphasis on systemic physiological mechanisms linking the musculoskeletal system to neuroendocrine, neurochemical, and neuroplastic regulation, as well as on the clinical implications for cognitive function, resilience, and stress tolerance.

A narrative approach was selected due to the multiscale complexity of the phenomenon under study, which involves dynamic interactions among biomechanical, metabolic, endocrine, immune, and brain processes. This type of review enables deeper conceptual integration and a pathophysiological discussion that would be difficult to achieve through an exclusively systematic or meta-analytic approach, particularly when the goal is to develop an integrative explanatory framework rather than estimate a single pooled effect size.

Although this review did not follow a formal PRISMA framework, efforts were made to enhance transparency and reproducibility by clearly describing the search strategy, eligibility criteria, and study selection process.

The literature search was conducted in international academic databases, including PubMed/MEDLINE, Scopus, and Web of Science. We prioritized English-language, peer-reviewed publications in high-impact international journals. Search strategies combined terms related to physical activity, exercise, and mental health—such as physical activity, exercise, depression, anxiety, stress, hypothalamic–pituitary–adrenal axis, neuroplasticity, inflammation, executive function, and cognition—using Boolean operators and adapting the approach to each database. The literature search was conducted between up to January 2026.

To improve transparency, eligibility criteria were defined *a priori* based on conceptual and methodological relevance. Studies were considered eligible if they (1): examined physical activity or structured exercise in relation to anxiety and/or depression (2); reported clinical, cognitive, or mechanistic outcomes relevant to stress regulation, neuroplasticity, or affective processes; and (3) were original studies, randomized controlled trials, or quantitative syntheses (systematic reviews or meta-analyses).

Exclusion criteria included studies lacking direct relevance to mental health outcomes, those focused exclusively on non-affective conditions, and publications without peer review.

Given the narrative and integrative purpose of this review, study selection also incorporated a theory-driven appraisal to ensure inclusion of conceptually informative and mechanistically relevant evidence.

We prioritized the inclusion of meta-analyses, systematic reviews, randomized controlled trials, and original mechanistic studies, including both human research and experimental models. To ensure clinical relevance and currency, articles published in the last ten years were preferred, with earlier seminal studies incorporated only when conceptually indispensable for understanding fundamental mechanisms.

Study selection was conducted in two stages. First, titles and abstracts were screened to identify potentially relevant publications. Second, full texts were assessed to determine final eligibility and conceptual contribution to the review framework. Evidence synthesis was qualitative and narrative, organized around thematic axes defined *a priori* that structure the manuscript. Discrepancies were resolved through discussion among authors.

## 3 Mechanics of movement and physical exertion as a primary biological stimulus

Physical activity encompasses any bodily movement produced by the musculoskeletal system that results in energy expenditure above resting levels and can be classified along a continuum of intensity ranging from sedentary to very vigorous. Within this broader construct, exercise represents a distinct subtype of physical activity characterized by planned, structured, and repetitive movements intentionally performed to improve or maintain one or more components of physical fitness, such as aerobic capacity, muscular strength, balance, agility, or coordination (8).

When viewed through a mechanistic lens, both physical activity and exercise can be conceptualized the organized application of mechanical forces to the musculoskeletal system, accompanied by coordinated increases in energy expenditure and neuromuscular activation. Each bout of movement involves the generation of muscle tension, deformation of biological structures, and load transmission across muscles, tendons, and bones. These forces constitute primary biological stimuli capable of triggering systemic adaptive cascades that extend beyond muscle tissue (9, 10).

From a physiological perspective, physical exertion represents a transient perturbation of homeostasis, characterized by metabolic shifts, autonomic activation, and neuroendocrine stimulation. Unlike chronic psychological stress, exercise-induced stress is predictable, titratable, and followed by recovery, which supports adaptive processes and allows it to be maintained within a “hormetic zone” in which beneficial systemic adaptations predominate (6, 7). In contrast, the chronic absence of this type of stimulus—characteristic of sedentary lifestyles—has been associated with greater vulnerability to mood disorders, cognitive decline, and reduced stress tolerance (11, 12).

In addition, repeated physical exertion can be interpreted as training for allostatic regulation, whereby the brain anticipates energetic and autonomic demands and more efficiently adjusts internal balance. Within this framework, interoception reflects bodily signals arising from these adjustments, and the discrepancy between what is anticipated and what is afferently signaled can be understood as a prediction error that updates internal regulatory models (13).

From a clinical-behavioral perspective, the graded physiological activation induced by exercise (e.g., increased heart rate and ventilation) can function as a form of interoceptive exposure: experiencing these sensations in a non-threatening context promotes greater tolerance and, with repetition, habituation to bodily cues that were previously feared, consistent with the principles of interoceptive exposure used in anxiety interventions (14–16).

In parallel, sustaining self-regulation under physiological load may support domains of cognitive control—such as inhibitory control and flexibility—that are sensitive to physiological state and relevant for modulating worry and rumination. Reviews on executive functions indicate that fitness and aerobic interventions are associated with modest but consistent improvements in these domains, albeit with methodological heterogeneity (17).

## 4 Musculoskeletal mechanotransduction and systemic signaling

Mechanical load and tension applied to skeletal muscle during exercise activate mechanotransduction processes, through which physical signals are converted into intracellular biochemical responses. These responses involve key signaling pathways such as mTORC1, MAPK/ERK, and AMPK, which integrate mechanical and energetic information to regulate protein synthesis, energy metabolism, and mitochondrial biogenesis—processes central to training adaptation and skeletal muscle tissue remodeling (9, 18).

**Figure 2:**
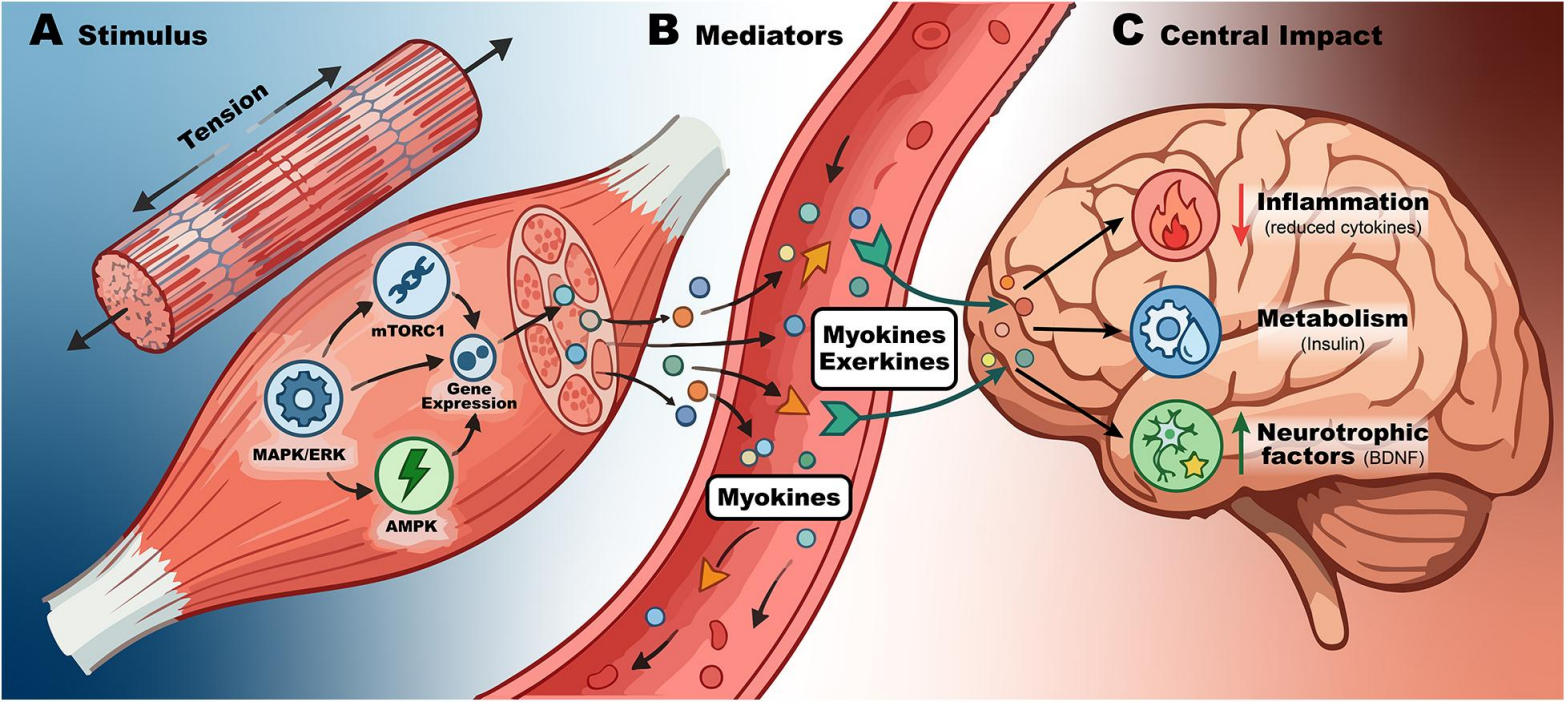
The muscle-brain axis: mechanotransduction and systemic signaling. Schematic representation of the concept of “biological transduction,” in which muscle contraction **(A)** induces the release of signaling molecules **(B)** that enter the circulation, cross the blood–brain barrier, and regulate inflammation and metabolism while promoting the expression of neurotrophic factors in the brain **(C).**

These adaptations are not limited to local structural changes; they also substantially reshape the muscle secretory profile, as skeletal muscle functions as a dynamic endocrine organ. During muscle contraction, multiple myokines and exerkines are released with autocrine, paracrine, and endocrine effects, capable of influencing distant tissues such as the liver, adipose tissue, the immune system, and the brain. Through this inter-organ communication, skeletal muscle establishes a functional muscle–brain axis (see Figure 2) that is fundamental for understanding the systemic effects of exercise on metabolism, inflammation, neuroendocrine regulation, and brain function (19, 20).

## 5 Metabolic regulation and modulation of the hypothalamic–pituitary–adrenal axis

Exercise consistently alters systemic energy metabolism, improves insulin sensitivity, and regulates substrate dynamics, which is associated with improved mood and reduced fatigue. From a neuroendocrine standpoint, exercise induces an acute activation of the hypothalamic–pituitary–adrenal (HPA) axis as part of an adaptive response to exertion, characterized by transient increases in cortisol and catecholamines. However, when the stimulus is repeated regularly and appropriately dosed, this activation is accompanied by improved regulatory efficiency of the axis, with more modulated hormonal responses and faster recovery in response to physical or psychosocial stressors (21–23).

This adaptation is particularly relevant in anxiety and depression, conditions in which HPA-axis hyperactivation or rigidity contributes to structural and functional alterations in brain regions involved in stress and emotion regulation, such as the hippocampus and prefrontal cortex. From this perspective, exercise can be interpreted as a form of stress-system training, capable of reducing accumulated allostatic load and promoting more flexible, adaptive responses to persistent environmental demands (3).

Beyond its affective effects, regulation of the HPA axis has direct implications for executive functions, domains that are especially sensitive to stress exposure. Experimental and clinical evidence indicates that chronic exposure to elevated glucocorticoid levels is associated with structural and functional alterations in the hippocampus and prefrontal cortex—regions that are critical for memory, executive control, and emotional regulation (3, 24). These changes are linked to impairments in processes such as working memory, inhibitory control, and cognitive flexibility, particularly under conditions of sustained stress (24).

In this context, the progressive normalization of HPA-axis reactivity induced by regular exercise may indirectly help preserve executive functioning by reducing allostatic load and mitigating the interference of chronic stress on prefrontal circuits, without implying a direct exercise–cognition causal relationship (3). From a functional standpoint, exercise can be cautiously conceptualized as “indirect cognitive training,” insofar as it exposes the organism to controlled physiological demands that require sustained self-regulation under neuroendocrine activation, thereby creating more favorable neurobiological conditions for executive performance (17). This framework is consistent with the observation that executive functions are highly sensitive to physiological state and stress, and that interventions that improve bodily regulation may translate into modest but clinically meaningful cognitive benefits (3, 17).

## 6 Anti-Inflammatory effects of exercise and their cerebral impact

A growing body of evidence indicates that a clinically relevant subgroup of individuals with depression—and in many cases with comorbid anxiety—exhibits low-grade systemic inflammation, characterized by persistent elevations in markers such as C-reactive protein, interleukin-6, and tumor necrosis factor alpha (25). Meta-analyses have documented increases in pro-inflammatory cytokines in major depression, which are associated with alterations in neurotransmission, tryptophan metabolism, and synaptic plasticity, thereby contributing to the persistence of affective and cognitive symptoms (26, 27).

Regular physical activity attenuates this inflammatory state through complementary mechanisms that include reductions in visceral adipose tissue, improvements in metabolic profile, and the release of myokines with anti-inflammatory effects. These adaptations reduce peripheral inflammatory signaling to the central nervous system and promote a brain milieu more conducive to emotional regulation and synaptic plasticity (28, 29). At the cerebral level, reduced microglial activation and an improved neurochemical environment contribute to better synaptic function and mood regulation (12, 30).

Moreover, this inflammatory signature has been linked to a profile of somatic and cognitive symptoms that includes fatigue, reduced energy, and disturbances in cognitive processes such as learning and memory, consistent with the cytokine-induced sickness behavior framework (30, 31). In population-based longitudinal studies, baseline levels of C-reactive protein and interleukin-6 have been prospectively associated with later onset of depression, particularly in relation to depressive cognitive symptoms rather than negative affect *per se* (32). Converging evidence from a large population cohort further shows that the combination of depression and elevated C-reactive protein levels was associated with poorer performance on a behavioral measure of executive function, supporting the notion that inflammation may contribute to residual cognitive symptoms in a clinically relevant subgroup of patients (33).

## 7 Exercise-Induced neurochemical modulation

Exercise consistently modulates several neurochemical systems involved in mood regulation, including serotonergic, dopaminergic, and noradrenergic neurotransmission, contributing to anxiolytic and antidepressant effects observed both acutely and chronically, particularly when the stimulus is embedded within sustained adaptive processes (34, 35).

In addition, the endocannabinoid system plays a relevant role in the response to exercise. Evidence synthesized through meta-analyses indicates that exercise alters circulating endocannabinoid concentrations, which has been linked to reduced anxiety, analgesia, and subjective feelings of well-being, thereby facilitating both emotional regulation and adherence to physical activity (36). These changes should be understood as facilitators of neuronal plasticity rather than as isolated mechanisms.

In parallel, exercise is associated with cerebral plasticity processes and executive functions—key domains for behavioral self-regulation and cognitive control—that are often impaired in depression and are related to rumination, cognitive slowing, and difficulties in decision-making (17).

Taken together, exercise-induced neurochemical changes should be interpreted not as isolated mechanisms, but as components of a permissive neurobiological environment that supports plasticity and the functional reorganization of circuits involved in emotional and cognitive regulation. Modulation of monoaminergic systems, together with the transient increase in circulating endocannabinoids induced by exercise, has been proposed as part of a broader set of signals that facilitate processes of brain adaptation, including mood regulation, motivation, and behavioral adherence (12, 34, 36, 37).

From this integrative perspective, exercise-induced neurochemical modulation functions as a scaffold that interacts with neuroendocrine and neurotrophic adaptations, setting the stage for self-regulation and cognitive control processes. This framework is consistent with evidence that executive functions are highly sensitive to physiological state and stress, and that interventions optimizing bodily regulation may indirectly enable modest but meaningful benefits in executive domains linked to rumination, decision-making, and resilience under stress (3, 17).

## 8 Neuroplasticity, cognitive function, and resilience

The convergence of mechanical, metabolic, endocrine, and immune signals induced by exercise promotes processes of structural and functional neuroplasticity. A central mediator of these changes is brain-derived neurotrophic factor, whose expression increases with regular physical activity and is associated with synaptogenesis, cerebral angiogenesis, and functional reorganization of frontolimbic circuits (37, 38).

Beyond neurotrophic changes, exercise has also been proposed as a modulator of additional plastic mechanisms, including adult neurogenesis (evidence primarily preclinical), which provides a plausible bridge between systemic adaptations induced by physical activity and cognitive benefits observed in some populations and models (39).

**Figure 3:**
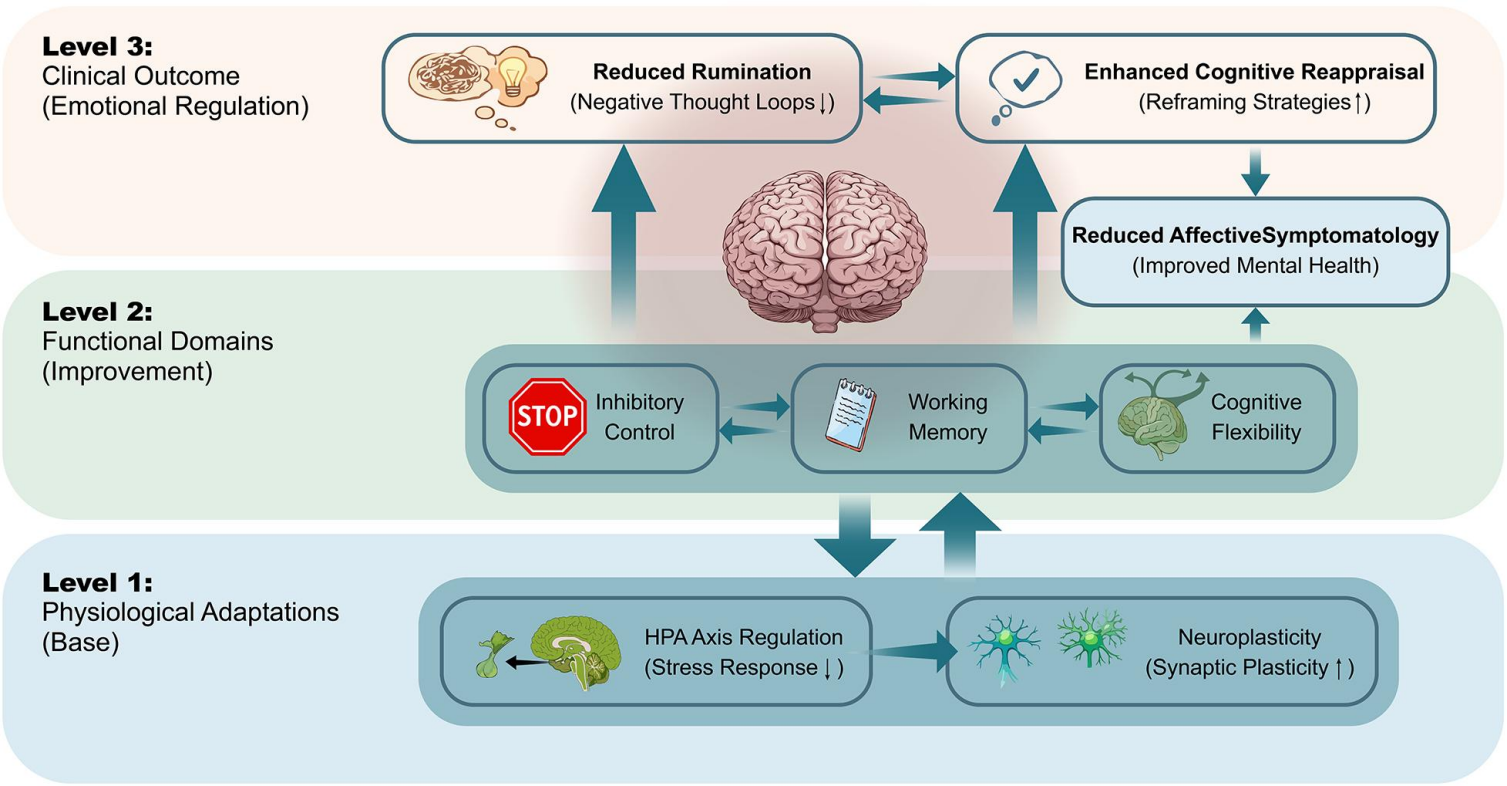
The cognitive node: integrative model of emotional regulation. Conceptual flow diagram illustrating the integrative model where physiological adaptations (Level 1) are proposed to relate to improvements in key executive functions (Level 2), which may facilitate emotional regulation through reduced rumination and improved cognitive reappraisal, and are associated with reduced affective symptomatology (Level 3).

These adaptations are particularly relevant in regions involved in stress regulation and executive functions, such as the hippocampus and prefrontal cortex. Improvements in inhibitory control, cognitive flexibility, and working memory may contribute to greater capacity for emotional regulation and adaptive coping, reducing patterns of rumination and emotional reactivity characteristic of anxiety and depression (17). Figure 3 illustrates an integrative conceptual model showing how physiological adaptations (Level 1) enhance executive functioning (Level 2), thereby promoting emotional regulation via reduced rumination and improved cognitive reappraisal, and leading to lower affective symptomatology (Level 3).

Taken together, these mechanisms allow physical exercise to be conceptualized as an integrated protective and therapeutic factor: it introduces a controlled physiological stressor, optimizes neuroendocrine regulation, reduces systemic inflammation, modulates central neurochemistry, and strengthens brain plasticity. The outcome is not only symptomatic reduction, but also an increase in neurobiological resilience, understood as the nervous system's capacity to adapt to and recover from adversity (3).

From a functional perspective, exercise-induced neuroplastic changes also affect domains of affective cognition, understood as the processes by which emotional information is perceived, evaluated, and regulated. Evidence indicates that cognitive flexibility and inhibitory control—functions dependent on prefrontal and frontolimbic circuits—play a key role in the capacity to modulate and disengage from patterns of repetitive negative thinking such as rumination, a central process in the vulnerability to and persistence of depression and anxiety (17, 40).

In this context, by strengthening the efficiency and plasticity of these circuits, exercise may facilitate more adaptive emotional regulation by reducing reactivity to negative stimuli and promoting more flexible cognitive reappraisal. These effects should be understood as the result of systemic adaptations that optimize the neurobiological environment for emotional self-regulation, rather than as a specific form of cognitive training, and they align with the concept of neurobiological resilience associated with improved stress regulation and lower allostatic load (3, 12).

To synthesize this integrative framework, Table 1 organizes the main proposed mechanistic axes through which exercise may influence cognitive-affective domains relevant to anxiety and depression. The table is not intended to establish univocal causal relationships or to rank mechanisms, but rather to map the convergence between biological adaptations (neuroplastic, neuroendocrine, immunometabolic, and neurochemical) and functional processes such as executive functions, affective cognition, and emotional regulation. This schema facilitates a comparative and cautious reading of the evidence, emphasizing that exercise effects are best understood as the product of interdependent adaptive cascades whose clinical impact may vary across individuals and subgroups (3, 12).

**Table 1:** Mechanistic axes of exercise and cognitive–affective domains.

Mechanistic axis activated by exercise	Key biological processes	Associated cognitive–affective domains	Clinical implication (cautious formulation)	Key references
Structural and functional neuroplasticity	Proposed neurotrophic/plastic changes (e.g., BDNF), synaptic/neuronal plasticity; vascular support/angiogenesis; possible contribution of adult neurogenesis (primarily translational evidence)	Memory; cognitive efficiency; executive functions	Provides a plausible neurobiological substrate for more efficient cognitive–emotional regulation	(12, 37–39)
Stress-axis regulation (HPA axis)	Allostatic framework: allostatic load and stress effects on the brain (conceptual)	Executive control under stress; emotion regulation	Consistent with a model in which interventions that improve stress regulation may promote greater adaptive flexibility	(3)
Reduction of systemic inflammation	Decreased pro-inflammatory signaling (“sickness behavior” framework); inflammation–symptom pathways (subgroups)	Fatigue/low energy; slowing; cognitive–somatic symptoms	Suggests a pathway through which reducing inflammation may support cognitive–affective domains in subgroups with elevated inflammation	(12, 30)
Neurochemical modulation	Changes in circulating endocannabinoids after exercise; monoaminergic changes as a general framework	Subjective well-being/affect; motivation; affect regulation	May facilitate adherence and modulate affective states; best interpreted as part of an adaptive cascade	(36, 37)
Strengthening of executive functions	Executive functions as domains (inhibitory control/flexibility/WM); rumination as a transdiagnostic process	Inhibitory control; cognitive flexibility; rumination/repetitive negative thinking	Compatible with a framework in which improvements in cognitive self-regulation support emotion regulation (without attributing specific causality to exercise in this row)	(17, 40)
Integrated neurobiological resilience	Resilience/allostasis: capacity to adapt and recover in the face of adversity (framework)	Adaptive coping; emotional stability	Integrative framework to describe sustained benefits when multiple physiological axes converge	(3, 12)

## 9 Clinical evidence for the effect of physical activity and exercise in depression and anxiety

To synthesize and contextualize the current evidence base, Tables 2, 3 summarize findings from systematic reviews and meta-analyses examining the associations between physical activity and exercise with depression and/or anxiety across different populations. Table 2 focuses on observational and interventional evidence related to habitual physical activity, including total and leisure-time activity and dose–response relationships, whereas Table 3 summarizes evidence from randomized controlled trials examining different types of exercise programs. Together, these tables provide an integrated overview of the direction, magnitude, and consistency of effects, while highlighting heterogeneity, population-specific patterns, and methodological limitations that inform cautious interpretation and clinical translation. Finally, current physical activity and exercise recommendations aimed at the prevention and treatment of depression and anxiety are presented to facilitate clinical application and to support health professionals in translating the available evidence into practical guidance.

**Table 2:** Effect of physical activity on depression and/or anxiety.

Outcome	Population	Physical activity	Results	Reference
Depression	Adults	Total physical activity	Higher physical activity volume was associated with a lower risk of depression in a nonlinear dose–response pattern. Physical activity may lower the risk of depression between 16% and 28% (4.4–17.5 mMET-h per week, respectively)	(41)
Depression	Adults	Leisure-time physical activity	Higher leisure-time physical activity was associated with a lower risk of incident depression in a nonlinear (U-shaped) dose–response relationship. Leisure-time physical activity associated with lower risk of depression (light: 27%, moderate: 17%, and high: 8%).	(42)
Depression	Youth, adults, older adults	Total physical activity	Higher physical activity was associated with a lower risk of incident depression across age groups. Associations were consistent across geographic regions and physical activity assessment methods.	(43)
Anxiety	Adults and older adults	Walking (moderate intensity) or aerobic + resistance exercise	No statistically significant reduction in anxiety symptoms.	(46)
Anxiety	Youth and adults	Total physical activity	Higher levels of physical activity were associated with lower risk of anxiety symptoms, any anxiety disorder, and generalized anxiety disorder.	(44)
Anxiety	Children, adolescents and adults	Leisure-time physical activity	Higher levels of leisure-time physical activity were associated with lower levels of anxiety symptoms. The effect was stronger for adults than for children and adolescents.	(45)
Anxiety	Children, adolsecents, and young adults	Aerobic, resistance, concurrent	Small to moderate reduction in state and trait anxiety.	(47)
Anxiety	Older adults	Aerobic, resistance, balance	Physical activity was associated with small-to-moderate reductions in anxiety symptoms. Larger effects for single-type PA (predominantly resistance training) and for interventions <12 weeks.	(48)
Depression and anxiety	Children and adolescents	Aerobic, resistance, concurrent	Concurrent training and moderate-intensity exercise showed the largest effects for depression, while resistance training and light-intensity exercise showed the largest effects for anxiety. Interventions <12 weeks were more effective for depression than ≥12 weeks.	(78)

**Table 3:** Effect of exercise on depression and/or anxiety.

Outcome	Population	Exercise intervention	Results	Reference
Depression	Adults	Aerobic, resistance, mind-body	Multiple exercise modalities are well tolerated and effective in reductions of depressive symptoms, principally when it is vigorous intensity, and it is equally effective for people with and without comorbidities.	(5)
Depression	Adults	Aerobic, resistance, concurrent	Reduced depressive symptoms with a moderate-to-large effect. Small and not significant effects when compared to psychological treatments or medication. Moderate effect of exercise as an adjunct to antidepressant medication.	(58)
Depression	Adults	Resistance	Moderate reduction in depressive symptoms, regardless of health status, volume, or strength gains	(49)
Depression	Adults	Concurrent	Reductions in depressive symptoms, mainly in middle-aged and older adults with depression and those with moderate depression. Moderate intensity, duration, and frequency training showed greater health benefits.	(55)
Depression	Older adults	Aerobic, resistance, mind-body	All modalities are equivalent in mitigating depressive symptoms	(79)
Depression	Older adults	Aerobic, resistance, concurrent, mind-body	All exercise modalities were associated with reductions in depressive symptoms. Resistance and mind–body exercise were the most effective type of exercise, followed by aerobic and concurrent training.	(54)
Depression	Adults and older adults	Resistance	Improved depressive symptoms in clinically diagnosed depressive adults that performed low and moderate-high intensity resistance training.	(51)
Depression	Youth	Aerobic, resistance, mind-body	All types of exercise were effective for elevated symptoms of depression. Resistance training were the better intervention for treatment and prevention of depression.	(50)
Anxiety	Children and adolescents	Aerobic, resistance, rehabilitation	Moderate reduction in anxiety symptoms. Larger effects for moderate- and high-intensity exercise.	(53)
Depression and anxiety	Adults	Aerobic, resistance, concurrent	Aerobic, resistance or concurrent training improves symptoms of depression and anxiety. Large effect for depression and moderate effect for anxiety.	(56)
Depression and anxiety	Adults	Aerobic	Moderate reduction in anxiety symptoms in people with a current diagnosis of anxiety and/ or stress-related disorders.	(52)
Depression and anxiety	Children, adolescents, and young adults	Aerobic, resistance, concurrent	Improvements in depressive and anxiety symptoms; moderate magnitude. Direction of effects was consistent, while magnitude varied by exercise type, age, and study design.	(57)

### 9.1 Effect of physical activity on depression and anxiety

The evidence summarized in Table 2 indicates that habitual physical activity is inversely associated with depression and, to a lesser extent, anxiety across the lifespan. Higher levels of total and leisure-time physical activity are consistently associated with a lower incidence of depression in adults, with similar inverse associations also observed in youth and older adults (41–43). These associations are particularly consistent in adult populations and at low-to-moderate physical activity volumes, where the greatest relative benefits are observed, while associations at higher physical activity volumes are more variable, reflecting the nonlinear and, in some cases, U-shaped dose–response patterns. In contrast, associations between physical activity and anxiety outcomes are less consistent, generally weaker, and more dependent on age group and physical activity domain (total or leisure-time).

Although the overall direction of evidence supports that physical activity may prevent or mitigate depressive and anxiety symptoms, substantial heterogeneity across studies [*I*^2^ range: 0%–74% (41, 42, 44, 45);] and the largely observational nature of the evidence limit certainty regarding the magnitude of effects and warrant cautious interpretation.

### 9.2 Effect of physical activity on depression

Evidence indicates that higher levels of total and leisure-time physical activity are consistently associated with reduced incidence of depression in adults, youth, and older adults (41–43).

Dose–response meta-analyses indicate that the association between physical activity volume and depression risk is not linear, such that the largest relative reductions in depression risk occur when individuals increase their physical activity from no activity to low or moderate levels, while additional increases in physical activity beyond these levels are associated with smaller incremental benefits. Specifically, total physical activity volumes between approximately 4.4 and 17.5 mMET-h/week were associated with 16%–28% lower depression risk, with diminishing additional benefits at higher volumes (41).

Leisure-time physical activity shows a similar nonlinear pattern, with light and moderate activity associated with greater relative risk reductions than very high volumes, consistent with a U-shaped association reported by Guo (42). Importantly, prospective cohort evidence including youth, adults, and older adults (43) indicates that higher physical activity is associated with lower incident depression, with consistent findings across geographical regions and physical activity assessment methods, suggesting that this inverse association is stable across populations and settings despite differences in study design.

### 9.3 Effect of physical activity on anxiety

In contrast, associations between physical activity and anxiety outcomes are more heterogeneous and population-dependent. In adults and older adults, moderate-intensity walking for the primary prevention of anxiety was not associated with a reduction in anxiety symptoms (46). However, broader observational evidence suggests that higher total physical activity is associated with lower risk of anxiety symptoms, any anxiety disorder, and generalized anxiety disorder in youth and adults, although dose–response patterns are inconsistent (44). Leisure-time physical activity is also associated with lower anxiety symptom levels across age groups, but effects appear stronger and more consistent in adults than in children and adolescents (45).

Additional evidence in children, adolescents, and young adults indicates small to moderate reductions in anxiety symptoms associated with aerobic, resistance, and concurrent physical activity, although effect sizes are modest (47). In older adults, physical activity (including aerobic, resistance, and balance activities) is associated with small to moderate reductions in anxiety symptoms, with larger effects observed for single-type activity (predominantly resistance-based) and shorter intervention durations (<12 weeks) (48).

### 9.4 Effect of exercise on depression and anxiety

Exercise interventions are associated with improvements in depressive and anxiety symptoms across the lifespan (Table 3). Across children, adolescents, adults, and older adults, resistance, aerobic, concurrent, and mind–body training modalities demonstrate beneficial associations with mental health outcomes. Resistance training shows the most consistent and strong associations with reductions in depressive symptoms across age groups, particularly in adults, older adults, and youth (49–51), while aerobic exercise demonstrates reliable benefits for anxiety outcomes, especially when prescribed at moderate to high intensities (52, 53). Concurrent and mind–body exercise are also effective but tend to show smaller or more variable effects compared with resistance-based interventions (5, 54). Despite the overall consistency in the direction of effects, the magnitude and certainty of these associations vary substantially by population, control group, exercise program design, health status, and study quality; therefore, while the evidence supports exercise as a relevant intervention for depression and anxiety, conclusions regarding the relative superiority of specific exercise modalities or optimal prescriptions remain tentative and context-dependent.

A central and consistent feature across all included reviews was the presence of substantial heterogeneity, with *I*^2^ values frequently exceeding 50% and, in several cases, surpassing 80%–90% (49, 51, 53, 55, 56). In parallel, many reviews reported high or unclear risk of bias, particularly related to blinding and allocation concealment. Together, these methodological limitations reduce confidence in the precision of pooled effect estimates and underscore the need for cautious interpretation of effect magnitudes, especially when comparing exercise modalities or translating findings into specific prescriptions.

### 9.5 Effect of exercise on depression

The evidence summarized in Table 3 indicates that exercise interventions are consistently associated with reductions in depressive symptoms across the lifespan, including youth, adults, and older adults. Across populations, multiple exercise types (resistance, aerobic, concurrent, and mind–body training) showed beneficial associations with depression outcomes, although the magnitude and certainty of effects vary by exercise type, control group, population characteristics and health status, and study design (5, 50, 54, 57).

The strongest and most consistent evidence for depression comes from adult populations. Network meta-analytic evidence indicates that aerobic, resistance, and mind–body exercise are all effective in reducing depressive symptoms, with larger effects observed when exercise is prescribed at higher intensities and with similar benefits reported for individuals with and without comorbidities (5). In Kvam (58), when compared with non-active controls, exercise demonstrates moderate-to-large reductions in depressive symptoms; however, effects are small and not statistically significant when compared directly with psychological treatments or antidepressant medication. Notably, exercise used as an adjunct to antidepressant medication confers a moderate additional benefit, suggesting that exercise effects are most pronounced when contrasted with minimal or usual care rather than with established first-line treatments.

Across age groups, resistance training has been examined in greater depth and shows particularly consistent associations with reductions in depressive symptoms. Evidence indicates moderate reductions in depressive symptoms that are largely independent of health status, training volume, or gains in muscular strength (49). Similar findings are observed in adults and older adults with clinically diagnosed depression, where both low- and moderate-to-high intensity resistance training are associated with improvements in depressive symptoms (51). In youth populations, all examined types of exercise are effective for elevated depressive symptoms, with resistance training emerging as the most effective modality for both treatment and prevention of depression (50). Evidence spanning children, adolescents, and young adults also indicates improvements in depressive symptoms following aerobic, resistance, and concurrent exercise, with moderate overall effects and variability in magnitude according to age and study design (57). Collectively, these findings support exercise (mainly resistance training) as a type of exercise for depression treatment across age groups, while acknowledging that effect estimates remain constrained by heterogeneity and methodological variability of studies.

### 9.6 Effect of exercise on anxiety

Exercise interventions are also associated with reductions in anxiety symptoms across youth and adult populations, although the magnitude of effects is generally smaller and more heterogeneous than those observed for depression. In children and adolescents, exercise interventions are associated with moderate reductions in anxiety symptoms, with larger effects observed for moderate- and high-intensity exercise (53). These findings suggest that exercise intensity may play a relevant role in anxiety-related outcomes in younger populations, although variability across interventions remains substantial.

In adults with diagnosed anxiety or stress-related disorders, aerobic exercise is associated with moderate reductions in anxiety symptoms when compared with non-active controls (52). In adults with depression and/or anxiety, combined evidence indicates that aerobic, resistance, and concurrent exercise are all associated with improvements in anxiety symptoms, although effect sizes are generally smaller than those observed for depression (56).

## 10 Physical activity and exercise recommendations to prevent or treat depression and anxiety

As previously mentioned, there is variability in the heterogeneity observed across the summarized studies in Tables 2, 3. This variability may be partly attributable to differences in physical activity recommendations and in the prescription of exercise components (frequency, intensity, time, and type).

There is no widely accepted consensus regarding the most effective exercise intervention for depression and/or anxiety, as exercise prescription necessarily depends on factors such as age group, health status, socioeconomic level, baseline activity level, and physical condition, among other characteristics. Nevertheless, the following section briefly outlines current recommendations for physical activity and exercise aimed at improving health outcomes and, more specifically, depression and anxiety.

Physical Activity Guidelines (59) indicate that regular moderate-to-vigorous physical activity reduces the risk of adverse health outcomes, including depression and anxiety. Adults should accumulate at least 150 min per week of moderate-intensity physical activity, 75 min per week of vigorous-intensity physical activity, or an equivalent combination, and perform muscle-strengthening exercises for all major muscle groups at least twice per week. Additional benefits occur with higher activity volumes (e.g., 300 min per week of moderate-intensity physical activity), and overall benefits generally outweigh the risks of injury. Older adults should engage in multicomponent activities that include balance, aerobic, and muscle-strengthening exercises, while children and adolescents should perform at least 60 min of moderate-to-vigorous physical activity daily.

Meanwhile, exercise guidelines (60) state that although high-quality scientific evidence remains limited, it is still possible to provide general recommendations for exercise to prevent and treat depression and anxiety. The following are exercise recommendations based on the FITT acronym for depression and anxiety:

Recommendations for aerobic exercise prescription for depression

- Frequency: At least 13 days (or longer) exercise programs. Cumulative exercise frequency appears to be more important for individuals with depressive disorders than for those without.
- Intensity: Physical activity at any intensity may reduce depressive symptoms, with stronger evidence for moderate-to-vigorous activity, and any amount of aerobic exercise is more beneficial than none.
- Time: Exercise produces acute improvements in affective states that can temporarily alleviate depressive symptoms. Bouts as short as 20 min may be sufficient for individuals without depressive disorders, whereas 45 min is recommended for those with depressive disorders.
- Type: Rhythmic aerobic exercises involving large muscle groups are recommended.

Recommendations for resistance exercise prescription for depression

- Frequency: At least two days per week is recommended, given the insufficient evidence to determine the optimal frequency for antidepressant effects.
- Intensity: Standard ACSM guidelines for safe muscle-strengthening exercise progression are recommended, given insufficient evidence to determine the optimal intensity for antidepressant effects.
- Time: Evidence is insufficient to determine the optimal resistance training duration for maximizing antidepressant effects
- Type: Both multi-joint and single-joint exercises targeting agonist and antagonist muscle groups are recommended, using a variety of exercise equipment and/or body weight.

Recommendations for flexibility exercise prescription for depression

- Frequency: Insufficient scientific evidence.
- Intensity: Insufficient scientific evidence.
- Time: Insufficient scientific evidence.
- Type: Stretching, meditation, and relaxation techniques can reduce depressive symptoms.

Recommendations for aerobic exercise prescription for anxiety

- Frequency: Three to five days per week.
- Intensity: Moderate-to-high intensity activities (60%–90% HRmax or ∼70% V˙O₂max) appear to be more effective; therefore, individually tailored programs that progress from walking to running, ideally with supervision, are recommended.
- Time: 20–60 min are effective, with greater benefits observed when progressing to longer sessions (60–90 min).
- Type: Rhythmic aerobic exercises involving large muscle groups are recommended.

Recommendations for resistance exercise prescription for anxiety

- Frequency: Two to three days per week.
- Intensity: Moderate-to-high intensity training based on perceived exertion scale (5–8 for beginners, and 6–10 for advanced exercisers); or one-repetition maximum (50%–70% for beginners, and 60%–85% for advanced exercisers).
- Time: Beginners (2–3 sets of 8–20 repetitions, and rest between sets 90–150 s); advanced exercisers (3–4 sets of 6–15 repetitions with self-selected rest)
- Type: Machines are recommended for beginners, while experienced exercisers may use machines or free weights; progression should move from smaller to larger muscle groups.

Recommendations for flexibility exercise prescription for anxiety

- Frequency: Three to five days per week.
- Intensity: Full-body movements involving extension, flexion, rotation, or stretching to the point of slight discomfort are recommended.
- Time: Static stretches should be held for 10–30 s, performed for 2–4 repetitions per exercise, for a total duration of at least 10 min per day.
- Type: Slow static stretching for all major muscle groups (e.g., yoga and Pilates).

**Figure 4:**
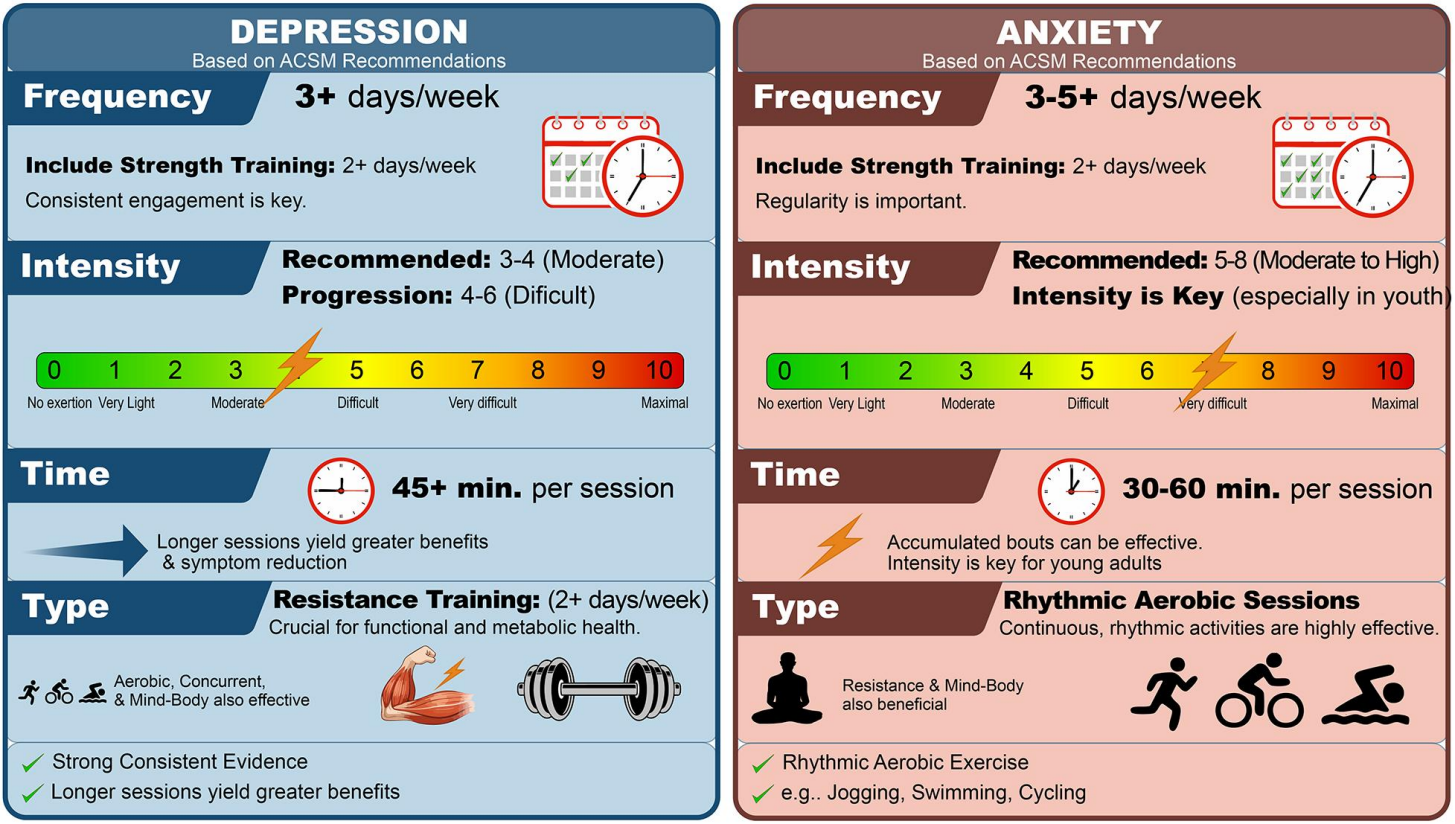
FITT prescription visual guide (frequency, intensity, time, type). Visual FITT prescription guide summarizing ACSM guidelines for Depression and Anxiety. This clinical tool facilitates personalized exercise prescription, highlighting key parameters for each condition to optimize mental health outcomes. FITT: Frequency, Intensity, Time, Type; RPE: Rating of Perceived Exertion.

Figure 4 provides a visual guide to the FITT principle (Frequency, Intensity, Time, and Type) based on the American College of Sports Medicine recommendations for counteracting symptoms associated with depression and anxiety (61–64).

## 11 Exercise, cognitive function, and depression

### 11.1 Exercise, cognitive function, and brain structure: clinical implications in depression

Beyond reducing affective symptoms, an increasingly clinically relevant issue is the impact of physical exercise on cognitive function and brain structure in people with depression. Depression involves not only emotional disturbances but also persistent deficits in attention, processing speed, memory, and executive functions, which are associated with poorer functional prognosis, reduced treatment response, and greater relapse risk—even when affective symptoms have improved.

Accumulated evidence indicates that exercise is associated with cognitive benefits across multiple domains, and the brain-health literature highlights favorable effects on cerebral perfusion, structure, and connectivity, particularly in regions involved in memory and executive control (37). In this line, a recent meta-analysis focused on adults with major depressive disorder reported that aerobic exercise is associated with significant improvements in global cognition, memory, and executive functions, with small-to-moderate effect sizes that are nonetheless clinically meaningful. Although modest in magnitude, these changes may facilitate emotion regulation, reduce rumination, and enhance the patient's capacity to benefit from psychotherapeutic interventions, reinforcing exercise as an integrative therapeutic strategy (65).

In parallel, additional plasticity-related mechanisms described in the translational literature—including adult neurogenesis modulated by physical activity—have been proposed as part of the biological substrate that could support cognitive benefits in certain contexts, although direct evidence in humans remains limited (39).

From a neurobiological perspective, the cognitive effects of exercise are accompanied by structural brain adaptations. The hippocampus—a region critical for memory and emotional regulation—is particularly vulnerable to both chronic stress and depressive states. A recent meta-analysis of randomized controlled trials in healthy older adults showed that aerobic training is associated with increases in hippocampal volume compared with control groups, suggesting that regular physical activity can induce measurable structural changes even in the absence of clinical pathology (66). These findings are especially relevant given that hippocampal atrophy has been consistently linked to allostatic load and stress-axis dysfunction in depression (3).

The cognitive and structural adaptations induced by exercise fit within a broader mechanistic framework that includes modulation of neuroplasticity, regulation of neurotrophic factors, improved cerebral blood flow, and reductions in neuroinflammatory processes. Together, these mechanisms create a brain environment more conducive to the maintenance and reorganization of circuits involved in emotion regulation and cognitive control.

In this context, incorporating explicit cognitive goals into exercise programs—for example, prioritizing aerobic modalities, coordinative-demand tasks, or progressions that challenge executive function—may be clinically pertinent, particularly for patients with depression who report prominent cognitive complaints. This evidence reinforces the value of exercise as an integrative therapeutic intervention whose benefits extend beyond symptom reduction to long-term brain and functional health.

## 12 Clinical integration: exercise as a component of a multimodal approach

Taken together, clinical evidence supports physical exercise as an effective, safe, and adaptable intervention for anxiety and depression. Its benefits include not only reductions in affective symptoms but also improvements in cognitive function and overall functional capacity. These effects position exercise as a valuable component within multimodal approaches that integrate psychotherapy, pharmacotherapy, and psychosocial strategies.

Within the pathophysiological framework developed in prior sections, exercise can be interpreted as an intervention that targets transdiagnostic processes, strengthening resilience to stress and reducing vulnerability to relapse. This conceptual integration supports a more systematic and mechanism-informed incorporation of exercise into mental health clinical practice (4, 12).

## 13 Exercise, weight regulation, and metabolic effects of psychopharmacological treatment

Weight gain and metabolic disturbances are clinically relevant adverse effects of psychopharmacological treatment in mood disorders. Among the agents with the greatest metabolic burden are several antipsychotics, with marked variability across molecules: in comparative meta-analyses, quetiapine shows evidence of weight gain and increased BMI, whereas olanzapine ranks among the least favorable profiles in terms of overall metabolic effects (Pillinger et al., 2020).

Although selective serotonin reuptake inhibitors are typically associated with more modest weight-related effects than other psychotropic medications, long-term population-based follow-up studies indicate that antidepressant treatment is associated with a sustained increase in the risk of ≥5% body-weight gain, peaking in the second and third year and remaining elevated for up to six years. This reinforces the need for weight-management strategies from early stages of treatment (67).

Treatment-associated weight gain may function as a negative clinical moderator in a context in which obesity and depression frequently co-occur and maintain bidirectional relationships; moreover, certain obesity-associated depressive profiles have been linked to symptoms such as hypersomnia and fatigue (68).

In this scenario, non-pharmacological interventions with lifestyle components (including diet and exercise) have shown efficacy in reducing or attenuating antipsychotic-induced weight gain, and prioritizing them—particularly in early treatment stages—has been recommended to favor preventive approaches (69).

## 14 Exercise, sleep, and circadian regulation in mood disorders

Sleep disturbance is a central and, in many cases, persistent component of mood disorders. In depression, continuity disturbances are frequently described—including sleep-onset insomnia, increased nocturnal awakenings, and early-morning awakening—alongside REM disinhibition characterized by shortened REM latency and changes in the organization of REM periods (70, 71).

Psychotropic medications can alleviate nocturnal symptoms but may also introduce physiological disruptions. In particular, several antidepressants—including SSRIs—are associated with insomnia/activation and REM suppression, which may translate into affective improvement with persistent or worsened sleep continuity in a subset of patients (71). By contrast, chronic use of sedatives/hypnotics has been linked to changes in sleep architecture and microstructure, with potential cognitive implications in vulnerable populations; therefore, symptomatic benefit does not necessarily equate to sustained neurocognitive restoration (72).

Within this framework, physical exercise emerges as a behavioral–physiological regulator of sleep. Quantitative evidence shows that acute exercise is associated with small but consistent effects on parameters such as sleep-onset latency, sleep efficiency, and wake time after sleep onset (73). In populations with insomnia, benefits appear more consistently in subjective sleep-quality outcomes (e.g., PSQI), whereas changes in objective measures such as efficiency or latency may be less robust or more heterogeneous depending on protocol and duration (74). In practice, exercise does not replace sleep medications in all cases, but it may support long-term goals by gradually improving sleep continuity and regulating the sleep–wake rhythm (75).

## 15 Discussion

The integrated evidence in this review consistently supports conceptualizing physical exercise as a systemic and transdiagnostic intervention capable of modulating core processes in the pathophysiology of anxiety and depression. Unlike therapeutic approaches targeting specific molecular pathways, exercise introduces a complex, multisystem physiological stimulus that reorganizes interactions among the musculoskeletal system, neuroendocrine axes, immunometabolic signaling, and brain function, with convergent effects on mood, cognition, and adaptive capacity in the face of stress (3, 12, 35).

Importantly, the relationships described in this framework should not be interpreted as unidirectional or strictly sequential. Instead, current evidence supports a multidirectional and interactive model in which peripheral, neurobiological, cognitive, and affective processes dynamically influence each other. In this context, current evidence supports a multidirectional and interactive model in which peripheral physiological adaptations, brain function, cognitive processes, and affective states dynamically influence each other, rather than following a unidirectional sequence.

Furthermore, while the proposed mechanisms are supported by a growing body of evidence, direct causal links between specific exercise-induced biological changes and clinical outcomes in humans remain only partially established. These considerations are consistent with the limitations summarized in Table 4, which highlight the gap between mechanistic plausibility and causal inference, as well as the influence of methodological heterogeneity on interpretation.

**Table 4:** Controversies and limits of the evidence.

Controversial aspect	What current evidence shows	Key limitation for clinical interpretation	Representative reference
Protocol heterogeneity	Aggregated evidence suggests average reductions in depressive symptoms, anxiety symptoms, and psychological distress with physical activity/exercise interventions.	Variation in modality, dose, supervision, comparators, and populations limits direct comparisons and fine-grained “prescriptions.”	(4, 5)
Mechanistic causality	Plausible mechanistic frameworks (stress/allostasis, inflammation, plasticity) are congruent with clinical effects.	Much mechanistic evidence is indirect or translational; the “dose → mechanism → clinical improvement” chain in humans is not fully established.	(12)
Interindividual variability in response	Average effects are consistent at the population level in high-level syntheses.	Moderators and response profiles (clinical/biological/contextual) remain poorly defined; study quality/heterogeneity limits personalized inferences.	(4, 5)
Anxiety vs. depression	Quantitative evidence is particularly robust for depression; for anxiety there is a signal of benefit, but with a smaller diagnosis-specific base.	Fewer RCTs by anxiety subtype and greater heterogeneity in outcomes/designs reduce diagnostic-level clinical precision.	(4, 77)
Cognitive outcomes	In MDD, a systematic review with meta-analysis focused on aerobic exercise suggests modest improvements in cognition.	Cognition is often a secondary endpoint; effect sizes are modest and sensitive to heterogeneity and measurement methods.	(65)
Translation to real-world clinical practice	In depression meta-analyses, structured/supervised programs tend to show larger effects than minimally supervised interventions.	Adherence, fatigue, access, comorbidities, and resources constrain sustained implementation and generalizability.	(76)

The evidence summarized includes quantitative syntheses (e.g., meta-analyses, network meta-analyses) and umbrella reviews; the reported effects reflect population-level averages. Protocol heterogeneity, interindividual variability, and the frequent treatment of cognition as a secondary outcome limit the precision of individualized recommendations and mechanistic causal inference (4, 5, 12).

From a contemporary pathophysiological perspective, both anxiety and depression can be understood as states of maladaptive dysregulation characterized by hyperreactivity of the hypothalamic–pituitary–adrenal (HPA) axis, low-grade systemic inflammation, alterations in neurotransmission, and progressive impairment of neuronal plasticity. Exercise appears to act directly and simultaneously on these domains, providing a robust mechanistic basis to explain its clinical efficacy observed consistently across randomized controlled trials and meta-analyses, as well as its capacity to enhance responses to pharmacological and psychotherapeutic treatments (4, 5, 76).

One of the most relevant conceptual contributions of exercise is its capacity to functionally modulate the HPA axis. Repeated exposure to a controlled physical stressor induces adaptations that improve regulatory efficiency of the stress system, reduce accumulated allostatic load, and promote more flexible hormonal responses to physical and psychosocial stressors. This adaptation is particularly relevant in anxiety and depression, where stress-axis rigidity is associated with symptom chronicity, cognitive vulnerability, and greater relapse risk (3, 21, 22).

Complementarily, exercise-induced reductions in low-grade systemic inflammation constitute a key mechanism of cerebral protection. Accumulating evidence indicates that inflammation interferes with monoaminergic neurotransmission, tryptophan metabolism, and synaptic plasticity—processes closely linked to anxious and depressive symptomatology. Exercise attenuates these inflammatory pathways through the release of anti-inflammatory myokines, reductions in visceral adipose tissue, and improvements in overall metabolic profiles, thereby creating a more favorable neurobiological environment for emotional regulation and cognitive function (26, 27, 29).

Within this framework, exercise-induced neurochemical modulation should be understood as part of an integrated adaptive cascade. Changes in monoaminergic systems, together with activation of the endocannabinoid system and regulation of neurotrophic factors, facilitate synaptic plasticity and functional reorganization of frontolimbic circuits—particularly in regions implicated in cognitive control and emotional regulation. These processes provide a neurobiological basis for sustained benefits of exercise on mood and cognition beyond transient or purely behavioral effects (34, 35, 38).

**Figure 5:**
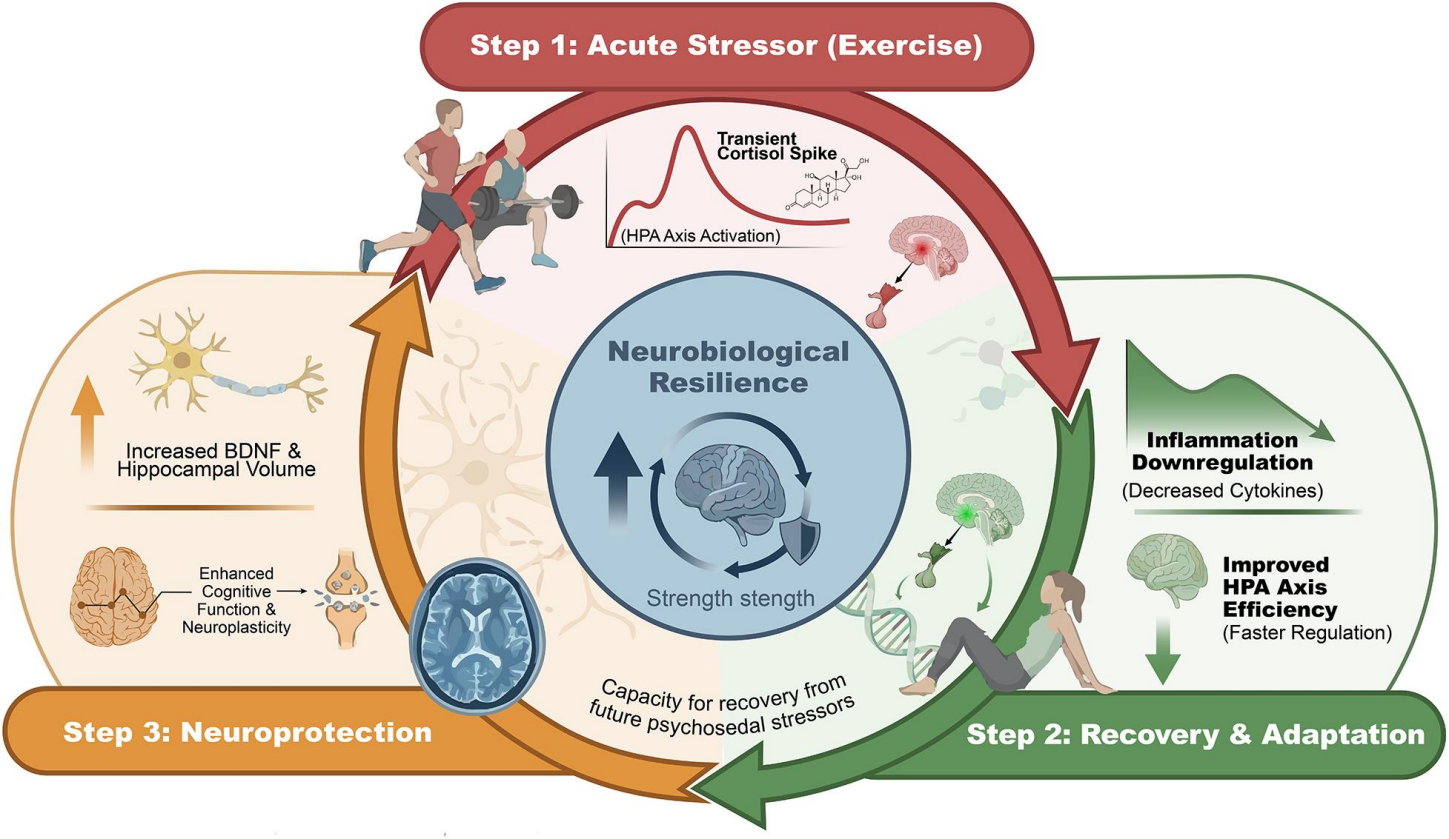
The neurobiological resilience cycle. Circular diagram illustrating the “Allostatic Load” model, where exercise acts as an acute stressor (Step 1), leading to physiological recovery and adaptation (Step 2), and culminating in long-term neuroprotection (Step 3), thereby enhancing overall neurobiological resilience and the ability to cope with future psychosocial stressors.

Improvements in executive functions emerge as a critical convergence point between the physiological mechanisms engaged by exercise and the observed clinical outcomes. Strengthening inhibitory control, cognitive flexibility, and working memory contributes to greater emotional regulation capacity, reduced rumination, and more adaptive coping with stress. From this perspective, exercise not only reduces affective symptoms but also strengthens neurobiological resilience, understood as the capacity of the nervous system to reorganize, adapt, and recover in the face of adversity (3, 17, 65). Figure 5 illustrates how the allostatic load model supports the cycle of neurobiological resilience, in which exercise acts as an acute stressor that induces physiological recovery and adaptation, ultimately leading to long-term neuroprotection and enhanced capacity to cope with future psychosocial stressors.

From a clinical standpoint, these findings support integrating physical exercise as a structural component of multimodal approaches to anxiety and depression. Evidence suggests that its prescription should be individualized—considering modality, intensity, duration, progression, and patient preferences—to maximize adherence and sustained benefits. This approach is especially relevant in contexts of chronic stress, high emotional burden, and metabolic comorbidity, where exclusively pharmacological interventions may be insufficient (4, 5, 76).

Finally, despite the strength of the available evidence base, important limitations remain, including methodological heterogeneity, variability in exercise protocols, and the lack of clinically actionable biomarkers to guide personalized prescription. Future studies should systematically integrate physiological, neurocognitive, and behavioral measures to identify response profiles, optimize dose and modality parameters, and consolidate exercise as a mechanism-informed therapeutic intervention within clinical mental health practice (4, 5, 12). Additionally, the narrative nature of this review implies that the literature search and study selection were not fully systematic or exhaustive. Although efforts were made to prioritize high-quality and conceptually relevant evidence, this approach may introduce selection bias and limit reproducibility.

Overall, the evidence synthesized in this review supports considering physical exercise as an intervention with multisystem biological effects relevant to the pathophysiology of anxiety and depression, beyond its traditional characterization as a behavioral strategy. Available studies indicate that exercise induces coordinated adaptations across neuroendocrine, immunometabolic, and neural systems, providing a plausible framework to explain its sustained effects on mood, cognition, and stress regulation (3, 12).

From this perspective, exercise can be understood as a dosable and adaptable intervention whose clinical implementation should be conceived as complementary to established pharmacological and psychotherapeutic treatments. Comparative evidence suggests that exercise can be safely and effectively integrated as part of multimodal approaches, with the potential to contribute to maintaining therapeutic benefits and improving functional outcomes depending on patient profiles and preferences. In this framework, recognizing the biological mechanisms of exercise does not imply establishing therapeutic hierarchies, but rather expanding the tools available for more personalized and sustainable mental health care (3, 5, 12).

To close this discussion with a clinically useful takeaway, it is important to emphasize that although the synthesized evidence—including meta-analyses, network meta-analyses, and umbrella reviews—supports average benefits of exercise for depression and anxiety, translating these effects to individual patients depends on sources of heterogeneity that remain incompletely resolved (e.g., variation in dose, modality, supervision, comparators, and clinical profiles), as well as the gap between plausible mechanistic models and direct causal evidence in humans. In this context, Table 4 concisely summarizes the main controversies and interpretive limits (protocol heterogeneity, interindividual variability in response, differences between depression and anxiety, cognitive outcomes often treated as secondary endpoints, and real-world implementation challenges) to support a prudent reading of the evidence and highlight priorities for future research and individualized prescription (4, 5, 12, 65, 76, 77).

## 16 Conclusions

The evidence integrated in this review positions physical exercise as a systemic biological intervention capable of meaningfully modulating the pathophysiology of anxiety and depression. Its effects on stress regulation, systemic inflammation, neuroplasticity, and cognitive function provide a solid basis for understanding exercise both as a protective factor and as a therapeutic tool.

Beyond symptom reduction, exercise strengthens neurobiological resilience and stress tolerance, supporting better adaptation to persistent physical and psychosocial demands. Integrating exercise into clinical practice represents an opportunity to advance toward more integrative, personalized, and sustainable models of mental health care.
